# Cardiovascular autonomic neuropathy in diabetes: an update with a focus on management

**DOI:** 10.1007/s00125-024-06242-0

**Published:** 2024-08-09

**Authors:** Aikaterini Eleftheriadou, Vincenza Spallone, Abd A. Tahrani, Uazman Alam

**Affiliations:** 1 Department of Cardiovascular and Metabolic Medicine, Institute of Life Course and Medical Sciences, University of Liverpool, Liverpool, UK; 2 Endocrinology, Department of Systems Medicine, University of Rome Tor Vergata, Rome, Italy; 3 Institute of Metabolism and Systems, School of Clinical and Experimental Medicine, University of Birmingham, Birmingham, UK; 4 Department of Diabetes and Endocrinology, Birmingham Heartlands Hospital, Birmingham, UK; 5 Liverpool Centre for Cardiovascular Science at University of Liverpool, Liverpool John Moores University and Liverpool Heart & Chest Hospital, Liverpool, UK; 6 Department of Medicine, University Hospital Aintree, Liverpool University Hospitals NHS Foundation Trust, Liverpool, UK; 7 Centre for Biomechanics and Rehabilitation Technologies, Staffordshire University, Stoke-on-Trent, UK

**Keywords:** Cardiac autonomic neuropathy, Diabetes, Diagnosis, Management, Pathophysiology, Review

## Abstract

Cardiovascular autonomic neuropathy (CAN) is an under-recognised yet highly prevalent microvascular complication of diabetes. CAN affects approximately 20% of people with diabetes, with recent studies highlighting the presence of CAN in prediabetes (impaired glucose tolerance and/or impaired fasting glucose), indicating early involvement of the autonomic nervous system. Understanding of the pathophysiology of CAN continues to evolve, with emerging evidence supporting a potential link between lipid metabolites, mitochondrial dysfunction and genetics. Recent advancements, such as streamlining CAN detection through wearable devices and monitoring of heart rate variability, present simplified and cost-effective approaches for early CAN detection. Further research on the optimal use of the extensive data provided by such devices is required. Despite the lack of specific pharmacological interventions targeting the underlying pathophysiology of autonomic neuropathy, several studies have suggested a favourable impact of newer glucose-lowering agents, such as sodium–glucose cotransporter 2 inhibitors and glucagon-like peptide-1 receptor agonists, where there is a wealth of clinical trial data on the prevention of cardiovascular events. This review delves into recent developments in the area of CAN, with emphasis on practical guidance to recognise and manage this underdiagnosed condition, which significantly increases the risk of cardiovascular events and mortality in diabetes.

**Graphical Abstract:**

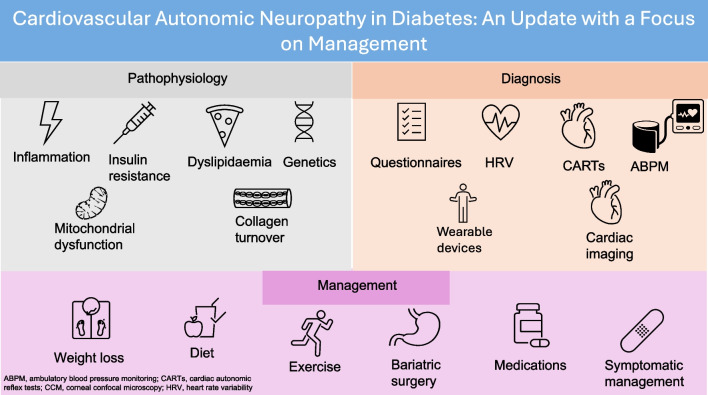

**Supplementary Information:**

The online version contains a slideset of the figures for download available at 10.1007/s00125-024-06242-0.

## Introduction

Cardiovascular autonomic neuropathy (CAN) is a highly prevalent microvascular complication of diabetes and results in dysfunction of the cardiovascular autonomic nervous system [1]. Dysglycaemia and metabolic derangements are the key precipitating factors in the development of CAN; however, the pathophysiological process differs between type 1 diabetes, which predominantly involves hyperglycaemia-related cellular mechanisms, and type 2 diabetes, in which insulin resistance and metabolic syndrome constituents have a complex relationship with developing CAN [2]. Importantly, the presence of CAN has been reported in prediabetes (impaired glucose tolerance and/or impaired fasting glucose) and the metabolic syndrome, demonstrating the importance of metabolic derangements in the pathogenesis of CAN, even in the absence of diabetes [3].

CAN is asymptomatic in the early stage of development; when symptoms occur, they are often non-specific and indicative of advanced disease [1]. Advanced CAN has been shown to be associated with a 5 year mortality rate of 25–50% [4], which is far in excess of that for both prostate cancer and breast cancer (~10%) [5, 6]. Optimising glycaemic management and CVD risk factors through targeted interventions is of paramount importance, as this has been shown to prevent the development of CAN and slow its progression to more advanced stages [7].

The prevalence of CAN is expected to rise in parallel with the increasing burden of type 1 and type 2 diabetes. Strategies are required for clinicians and health professionals to manage patients adequately. In this review, we aim to provide an up-to-date clinical reference for CAN, for healthcare professionals practising in diabetes, with a focus on management in the real-world setting.

## Epidemiology of CAN

There is wide variability in the reported prevalence of CAN because of the heterogeneous populations that have been studied and the diverse definitions of CAN used. The risk of developing CAN increases with age, duration of diabetes and suboptimal glycaemic/metabolic control [8]. Insulin resistance, obesity and CVD, as well as polycystic ovarian disease and hepatic steatosis, have also been suggested as risk factors for the development of CAN [2]. Ethnic variations in risk and prevalence of CAN have been demonstrated; in particular, men of African descent have increased sympathetic nervous system activity that is independent of weight, and this may contribute to early or more pronounced CAN signs and symptoms, as this first impairs the parasympathetic system activity [9].

Prevalence figures vary according to the aetiology of diabetes, with prevalence reported as being between 29% and 54% for type 1 diabetes and between 12% and 73% for type 2 diabetes (Table 1). The recent study reported by Davis et al indicates that, among participants with type 2 diabetes, the prevalence of possible CAN is 33.7%, while the prevalence of definite CAN is 15.3% [10]. In a large population-based study including 3010 participants with type 1 diabetes, the prevalence of possible CAN was found to be 36% [11]. Low et al reported a prevalence of definite CAN of 54% among a smaller cohort of participants with type 1 diabetes (*n*=83) [12]. Eleftheriadou et al demonstrated through a comprehensive systematic review that CAN prevalence is higher in those with prediabetes (9–38%) than in those with normal glucose tolerance (0–18%) [3]. The KORA S4 survey determined the prevalence of CAN in a population with prediabetes and demonstrated increasing prevalence with increasing severity of dysglycaemia: 4.5% for normoglycaemia, 5.9% for impaired glucose tolerance, 8.1% for impaired fasting plasma glucose and 11.4% for a combination of impaired fasting plasma glucose and impaired glucose tolerance [13].

**Table 1:** CAN prevalence in type 1 and type 2 diabetes in clinic- and population-based studies

Author, year, country and study type	Type of diabetes	Sample size	No. with diabetes	Age (years)^a^	Diabetes duration (years)^a^	Diagnostic test used	No. of AFTs used for diagnosis	Prevalence (%)
Davis et al [10], 2024, Australia, population-based study	T2D	830	830	62.3 ± 10.5	7.1 (3.0–15.0)	HRV during deep breathing, the Valsalva manoeuvre and on standing	Possible CAN: 1 Definite CAN: ≥2	Possible CAN: 33.7 Definite CAN: 15.3
Dimova et al [104], 2017, Bulgaria, clinic-based study	T2D	478	121	54.4 ± 11.5	0 (newly diagnosed)	HRV at rest and during deep breathing, Valsalva challenge and standing challenge	≥2	32.2
Lerner et al [105], 2015, Peru, clinic- and population-based study	T2D	384	213	57.6 ± 8.3	NA	Valsalva manoeuvre, BP postural decrease, 30:15 ratio, E:I ratio	≥2	37
Ziegler et al [13], 2015, Germany, population-based study	T2D	1332	n-DM:78 k-DM:130	n-DM: 66 (61–71) k-DM: 65 (61–69)	n-DM: 0 k-DM: NA	Linear HRV analysis and non-linear HRV analysis derived from non-linear dynamics	≥2	n-DM: 11.7 k-DM: 17.5
Tahrani et al [30], 2014, UK, clinic-based study	T2D	204	204	56.43 ± 12.07^b^	12.40 ± 7.68^b^	E:I ratio, Valsalva manoeuvre, 30:15 ratio, BP postural decrease	≥2	42.2
Pop-Busui [106], 2009, USA, clinic-based study	T1D	1211	Intensive treatment group: 620 Conventional treatment group: 591	Intensive treatment group: 47.8 ± 7.0 Conventional treatment group: 47.0 ± 6.9	Intensive treatment group: 26.6 ± 4.9 Conventional treatment group: 26.1 ± 4.9	E:I ratio, Valsalva manoeuvre, DBP postural decrease	≥2	Intensive treatment group: 29 Conventional treatment group: 35
Wu et al [107], 2009, China, population-based study	T2D	1638	157	57.7 ± 12.8	NA	OH, BP and HRV after standing	1	25.5
Low et al [12], 2004, USA, population-based study	T1D and T2D	476	T1D: 83 T2D: 148	T1D: 50.9 ± 14.7 T2D: 64.1 ± 11.7	T1D: 24.3 ± 11.1 T2D: 15.3 ± 8.6	CASS, sudomotor axon reflex test, HRV during Valsalva manoeuvre, SBP postural decrease, 30:15 ratio, E:I ratio	≥2	T1D: 54 T2D: 73
Valensi et al [108], 2003, France, clinic-based study	T1D and T2D	396	T1D: 245 T2D: 151	T1D: 36.1 ± 0.8 T2D: 45.5 ± 0.8	T1D: 10.0 ± 0.6 T2D: 6.3 ± 0.5	HRV during: deep breathing, Valsalva manoeuvre and lying-to-standing tests	1	T1D: 54.1 T2D: 45.4
Kempler [11], 2002, Europe, population-based study	T1D	3010	3010	32.7 ± 10.2	14.7 ± 9.3	30:15 ratio, SBP postural decrease	1	36

^a^Data are mean (± SD or range)

^b^The mean ± SD for all participants was calculated based on the data provided for the two groups (CAN and no CAN). These values were not directly provided in the study

AFT, autonomic function test; CASS, Composite Autonomic Severity Score; DBP, diastolic BP; E:I ratio, expiration/inspiration ratio; HRV, heart rate variability; k-DM, known diabetes mellitus; n-DM, newly diagnosed diabetes mellitus; NA, not available; OH, orthostatic hypotension; SBP, systolic BP; T1D, type 1 diabetes; T2D, type 2 diabetes; 30:15 ratio, heart rate response to standing

## Pathophysiology

**Fig. 1:**
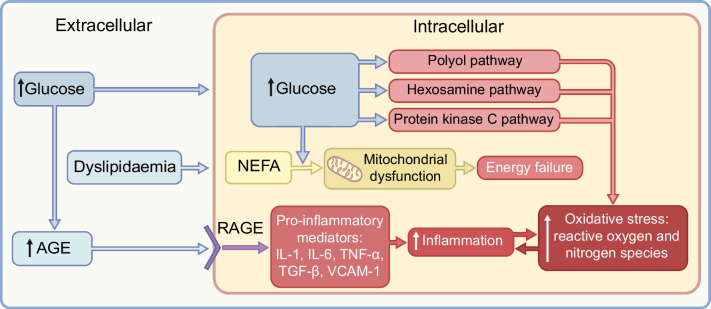
Pathophysiology of diabetic autonomic neuropathy: the role of hyperglycaemia and dyslipidaemia. Hyperglycaemia and dyslipidaemia contribute to increased inflammation, oxidative stress and energy failure in autonomic neurons, ultimately leading to autonomic dysfunction [14, 15, 17]. VCAM-1, vascular cell adhesion molecule 1; RAGE, AGE receptors. This figure is available as part of a downloadable slideset

The precise pathophysiology of CAN remains unclear; however, multiple pathways leading to microvascular complications have been identified: AGE-mediated inflammation, increased oxidative stress and reactive oxygen species causing direct nerve cell damage, activation of the hexosamine, protein kinase C and polyol pathways and subsequent osmotic and oxidative stress, and neuronal ischaemia due to diabetic microangiopathy (Fig. 1) [14–16]. Insulin resistance, elevated BMI, increased waist circumference and hypertension are associated with abnormal autonomic indices. This is primarily due to hyperinsulinaemia-driven activation of the sympathetic nervous system and parasympathetic impairment, resulting in sympathetic predominance. This sympathetic predominance can further exacerbate insulin resistance and hyperinsulinaemia, creating a vicious cycle [2].

More recently, mitochondrial dysfunction [17], lipid metabolites in early-onset type 2 diabetes [18] and collagen turnover in type 1 diabetes [19] have been investigated and are emerging pathophysiological pathways in diabetic autonomic neuropathy. Mitochondrial dysfunction due to high glucose and lipid levels impairs distal nerve fibres, primarily because of anatomical challenges (Fig. 1). The disruption of mitochondrial transport in long, narrow axons leads to energy failure and axonal injury [17]. High-fat diets and fatty acid chain length impact neuronal mitochondria, suggesting a potential role of dietary interventions to mitigate neuropathy, particularly in type 2 diabetes [20]. The ADDITION study noted an association between high plasma triglyceride levels and CAN, although this association decreased over time [18]. Ziegler et al showed that specific lipid metabolites (phosphatidylcholines [five diacyl and one acyl-alkyl] and sphingomyelins [C16:0 and C16:1]) are linked to cardiac autonomic dysfunction in individuals recently diagnosed with type 2 diabetes, indicating a potential role for disrupted lipid metabolism in the early development of CAN [21]. Hansen et al reported that increased collagen turnover (specifically collagen 3 and 6 turnover markers) is associated with CAN, which may reflect heightened fibrosis and could affect both parasympathetic and sympathetic function, although whether it is a cause or a result of nerve damage remains unclear [19].

Genetic susceptibility may play a role in the development of diabetic neuropathy, with several genes identified as being involved in endothelial dysfunction (*ACE*), oxidative nitrosative stress (*MTHFR*, *GPX1* and *CAT*) and lipid metabolism (*TCF7L2*) [22, 23]. Specific SNPs of miRNA-related genes have been linked to CAN risk in type 2 diabetes [1]. The C allele of rs2910164 in *MIR146A* was associated with reduced CAN risk, while the variant allele of rs895819 in *MIR27A* was linked to higher early CAN risk [24]. Additionally, the *MIR499A* GG genotype, along with disease duration and HbA_1c_ level, was independently associated with early CAN. However, a previous study in twins without diabetes reported the absence of a genetic influence on cardiovascular autonomic function after adjusting for multiple variables in the heritability estimates [25].

## Clinical manifestations of CAN

**Fig. 2:**
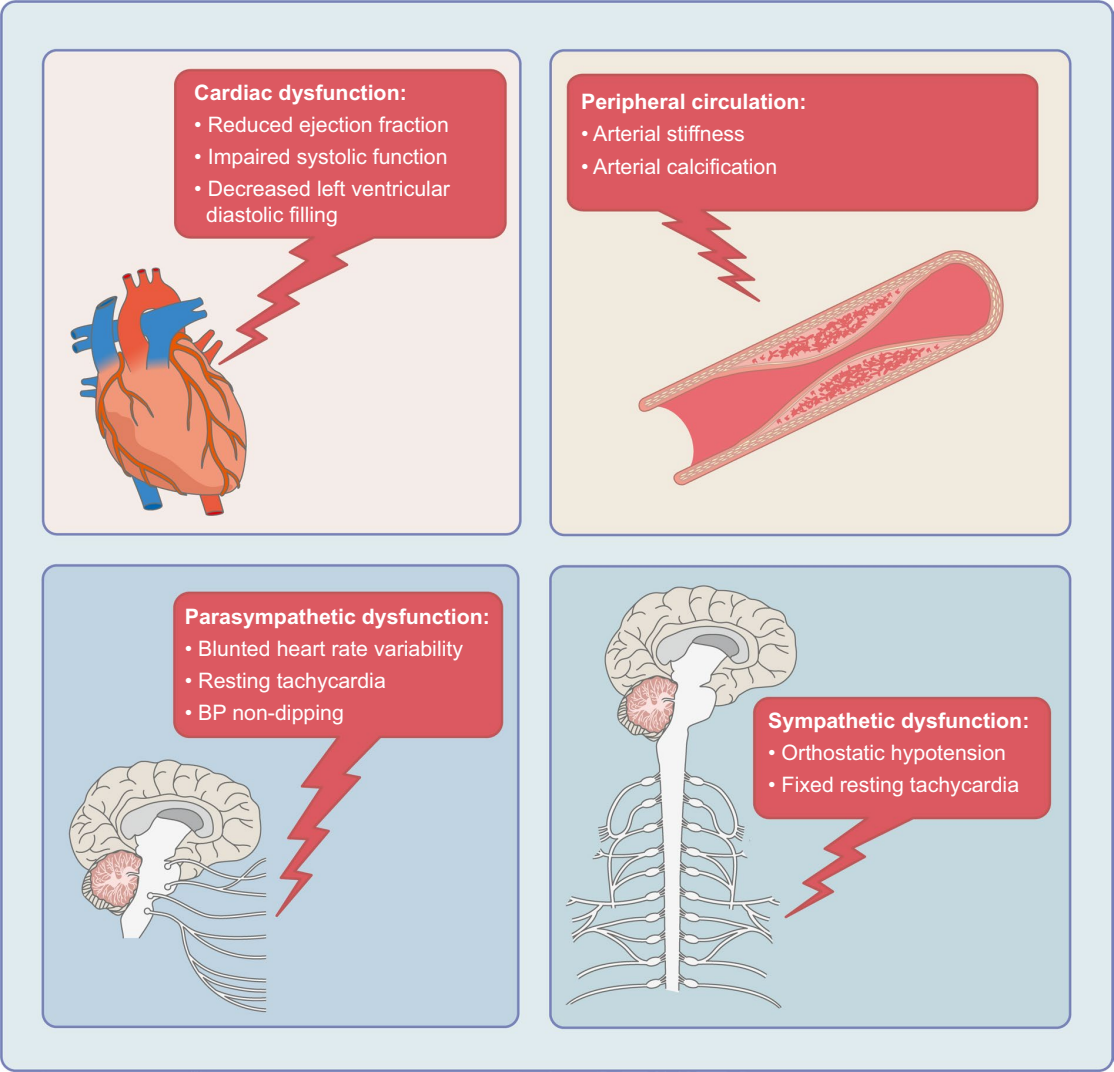
Clinical presentations of CAN. Clinical manifestations of CAN that occur because of parasympathetic dysfunction, cardiac dysfunction, peripheral circulation issues and eventually sympathetic dysfunction are shown [4, 26]. The parasympathetic system is affected first, leading to sympathetic predominance, which manifests as resting tachycardia. As CAN progresses, the sympathetic system also becomes involved, which leads to advanced signs/symptoms of CAN such as orthostatic hypotension. This figure is available as part of a downloadable slideset

CAN is characterised by a range of clinical manifestations, with subtle and insidious onset of symptoms at various stages of its natural history (Fig. 2) [1]. The progression of damage to the nervous system follows an inverse relationship with nerve length, with the longest autonomic nerve, namely the vagus nerve, affected first [4]. Early disease leads to dysfunction in the parasympathetic system and consequently sympathetic predominance. Clinically, this presents with resting tachycardia (up to 130 beats/min). Advanced CAN leads to a fixed heart rate with inability to appropriately respond to physiological stressors, for example exercise. This attenuated response presents clinically as exercise intolerance.

In the advanced stages of CAN, orthostatic hypotension emerges, presenting in symptomatic forms as dizziness, light-headedness or even syncope on standing. Orthostatic hypotension is defined as a sustained reduction in systolic BP of ≥20 mmHg (≥30 mmHg in the presence of supine hypertension) or in diastolic BP of ≥10 mmHg on transitioning from a supine to a standing position [26]. Baroreflex impairment and sympathetic denervation result in failure of an increase in sympathetic outflow to vessels and peripheral vascular resistance to counteract the reduction in venous return. In the ACCORD study, orthostatic hypotension was independently correlated with an increased risk of total mortality (HR 1.61, 95% CI 1.11, 2.36) and heart failure death or hospitalisation (HR 1.85, 95% CI 1.17, 2.93) [27]. Treatment is indicated only for symptomatic forms with the aim of minimising symptoms while not exacerbating supine hypertension. Non-pharmacological interventions include elastic stockings (full legs), abdominal binder, bed tilt-up if not contraindicated and physical counter-manoeuvres. Midodrine and droxidopa are the only drugs approved by the US Food and Drug Administration [23].

A higher prevalence of silent myocardial ischaemia among individuals with CAN has been reported. The pooled prevalence rate risk of silent myocardial ischaemia in a meta-analysis was 1.96 (95% CI 1.53, 2.51, *n*=1468), indicating a consistent association with CAN [28]. A recent prospective cohort analysis of the ACCORD study revealed a 1.9‐fold greater risk of silent myocardial ischaemia among participants with CAN compared with those without CAN, as well as a positive association between CAN and an increased risk of silent myocardial ischaemia when accounting for several known confounders [29]. Tahrani et al documented a significant independent association between CAN and various renal complications including chronic kidney disease, albuminuria and eGFR decline among individuals diagnosed with type 2 diabetes [30].

## Association of CAN with hypoglycaemia and excess cardiovascular and mortality risk

Although the potential pathways relating CAN to severe hypoglycaemia are not fully understood, a recent post hoc analysis of the ACCORD study highlighted that CAN represents a significant independent factor associated with heightened vulnerability to both first and recurrent severe hypoglycaemic episodes among adults diagnosed with type 2 diabetes [31]. However, as emphasised in the ADA Standards of care in diabetes, although impaired counterregulatory responses to hypoglycaemia in both type 1 and type 2 diabetes can lead to hypoglycaemia unawareness, there is no direct link to autonomic neuropathy [32]. Moreover, Arshad et al demonstrated that CAN is not a primary factor in determining the reversibility of impaired hypoglycaemia awareness [33].

Hypoglycaemic episodes exacerbate the strain on an already impaired autonomic nervous system, prolonging the corrected QT interval (QTc) and contributing to adverse outcomes. The ACCORD study highlighted the previously unrecognised harm of intensive glucose lowering in individuals with type 2 diabetes [34]. Post hoc analysis  has since demonstrated that severe hypoglycaemia is associated with an increased risk of death, independent of the treatment arm (therapy for intensive glycaemia management vs therapy for standard glycaemia management) [34]. A recent meta-analysis demonstrated a 42% increase in the risk of arrhythmia, a 59% increase in the risk of death attributed to CVD and a 68% increase in the risk of all-cause mortality in individuals with hypoglycaemia compared with euglycaemic individuals [35]. Chow et al investigated the association between hypoglycaemia and cardiac arrhythmias through continuous glucose and ambulatory electrocardiographic monitoring [36]. They observed an increase in bradycardia (eightfold) and atrial (fourfold) and ventricular ectopic activity during hypoglycaemic periods (primarily nocturnal) and concluded that hypoglycaemia is a proarrhythmic condition.

The association between hypoglycaemia and cardiovascular risk is multifactorial [37]. Hypoglycaemia triggers heightened sympathetic activity, which impacts the cardiovascular system by increasing heart rate and systolic BP. Hypoglycaemia can directly suppress cardiomyocyte potassium currents and elevate intracellular calcium levels, predisposing individuals to QTc prolongation, ventricular tachycardia and ventricular fibrillation. Kacheva et al demonstrated a mean±SD increase in QTc from 415.1±21.9 ms at baseline to 444.9 ± 26.5 ms during induced hypoglycaemia in a population with normal baseline ECG, the majority of whom did not have diabetes or prediabetes (*n*=106/119) [38]. Moreover, Marques et al have shown that the degree of QTc prolongation during hypoglycaemia is greater in individuals with type 1 diabetes than in those with type 2 diabetes (QTc prolongation, median [range]: type 1 diabetes 156 [8–258] ms; type 2 diabetes 128 [16–166] ms) [39].

Overall, CAN diagnosis may help in tailoring the targets of glycaemic management in individuals with type 2 diabetes at higher cardiovascular risk [1]. Moreover, both acute and antecedent hypoglycaemia affect cardiovascular autonomic control, but if and how CAN exerts a modulatory effect on these responses has not been defined.

## Diagnosis

### Symptoms/signs

Screening for diabetic autonomic neuropathy should be based on careful clinical assessment of signs/symptoms [1]. Some questionnaires have been devised to capture autonomic symptoms, including the Autonomic Symptom Profile (ASP) and its simplified 84 question Composite Autonomic Symptom Score (COMPASS) and 31 question (COMPASS 31) versions, with COMPASS 31 validated for autonomic symptoms of diabetic neuropathy [40]. Such questionnaires have been developed to gauge the risk of CAN, aiding in the identification of individuals at elevated risk. However, notably, Low et al have shown a weakness in such symptom tools in mild diabetic neuropathy, as its correlation with deficit scores was weak overall, especially in type 2 diabetes [12]. Therefore, definitive diagnosis should continue to rely on cardiac autonomic reflex tests (CARTs), as specified in various guidelines [41–43] (Table 2).

**Table 2:** Recommended diagnostic testing for CAN in clinical practice and research according to different guidelines

CAN tests	ADA [42, 85]	Toronto Consensus [41]	American Autonomic Society, American Academy of Neurology and International Federation of Clinical Neurophysiology [109]^a^	American Association of Clinical Endocrinologists and American College of Endocrinology [43]
CAN tests for clinical diagnosis	• HRV with deep breathing^b^ • Resting tachycardia (>100 bpm)^b^ • Orthostatic hypotension test^b^	• Heart rate cardiovascular tests (gold standard) • Orthostatic hypotension test • QT interval (additional information and risk stratification) • ABPM for dipping status (early additional information and risk stratification) • HRV (early additional information and risk stratification)	• Cardiovascular adrenergic function: Valsalva manoeuvre and the tilt table test with continuous heart rate and beat-to-beat BP monitoring Cardiovagal function: HRV in response to breathing, Valsalva ratio, 30:15 ratio	• Resting heart rate • BP responses to standing • HRV in response to deep breathing, standing and Valsalva manoeuvre
CAN tests for research purposes	• Heart rate cardiovascular tests • HRV • Resting heart rate • QT interval	• Heart rate cardiovascular tests^c^ • Orthostatic hypotension test • QT interval • ABPM for dipping status • HRV^c^ • Baroreflex sensitivity measures^c^ • Scintigraphy studies^c^ • Muscle sympathetic nerve activity • Catecholamine assessment	Additional tests: • Cold pressor test/cold face test	Not mentioned

^a^Electrodiagnostic assessment

^b^In conjunction with symptoms

^c^Clinical trial endpoint recommendation

### Cardiac signs

Reduced heart rate variability (HRV), prolonged QT interval and resting tachycardia serve as vital indicators of CAN. HRV indices provide key information on both sympathetic and parasympathetic cardiac dysfunction [44]. Pop-Busui et al demonstrated fair agreement between time-domain HRV indices obtained from standard 10 s 12 lead ECG recordings and the established gold standard CARTs, supporting their potential utility as a measure of CAN [45]. Kempler et al suggested using HRV in combination with heart rate response from lying to standing as the optimal initial screening tool [11]. In this context, the focus on simplifying CARTs or substituting them with HRV indexes holds significant promise, including potential advancements such as the adoption of ultra-short ECG (less than 5 min duration)-derived measures. Nonetheless, it is crucial to approach this with caution, considering the ongoing debate regarding the reliability of ultra-short ECG recordings [46]. In a meta-analysis (*n*=4584 participants with diabetes), prolonged QT interval was shown to be a specific (86%) indicator of diabetic autonomic failure, but lacked sensitivity (28%) [47]. Resting tachycardia is a readily available, although not specific, measure of CAN and in the clinic can be used as an adjunct in identifying those with CAN [41].

### CARTs/ambulatory BP monitoring

CARTs were described in 1982 by Ewing and Clarke, and have been recommended as the reference standard method of diagnosis for CAN [1, 48]. Moreover, CARTs have emerged as a method for staging CAN: a singular abnormal test suggests potential or early-stage CAN and a minimum of two abnormal tests suggests definite CAN [41]. The presence of orthostatic hypotension alongside irregularities in heart rate tests signals severe or advanced-stage CAN. The diagnostic value of each CART has been a matter of debate in the literature, without a conclusive demonstration of superior diagnostic performance of any one test compared with any other [49, 50]. The role of ambulatory BP monitoring (ABPM) in the diagnosis of CAN has been explored, with the finding that a non-dipping or reverse dipping pattern is a marker of autonomic neuropathy with high specificity but low sensitivity [51].

### Early tests for CAN: baroreflex sensitivity and cardiac sympathetic imaging

Arterial baroreceptors regulate heart rate and arterial tone to stabilise BP (a decrease in arterial pressure results in decreased vagal and increased sympathetic outflow and vice versa). Baroreflex sensitivity can be measured (mainly in the vagal cardiac arm) and has been found to be impaired prior to the development of abnormal CARTs [23]. Radiolabelled sympathetic neurotransmitter analogues allow direct assessment of cardiac sympathetic innervation via myocardial scintigraphy, which has shown greater sensitivity in identifying sympathetic de-innervation than HRV analysis [52]. However, these modalities are primarily used in research because of their specialised and costly nature.

### Emerging diagnostic tests for CAN

Current wearable devices enable seamless, continuous and low-cost ECG monitoring, which provides information about cardiac features such as heart rate, HRV and QT intervals [53]. As highlighted by Daskalaki et al, these devices can continuously assess cardiac autonomic function; however, further studies are needed to determine how these data can best support early recognition of diabetic complications, including CAN [53]. Recent studies have revealed corneal nerve loss as an indicator of CAN, introducing a novel screening method using corneal confocal microscopy (CCM) [54]. Tavakoli et al demonstrated that CCM-detected corneal nerve damage effectively diagnoses both subclinical and overt diabetic autonomic neuropathy with high sensitivity and specificity [55]. CCM may overcome the limitations of other diagnostic methods that are affected by cardiovascular comorbidities and medications [56] and the need for physical manoeuvres.

## Management

### Glycaemic vs multifaceted approaches

Both the DCCT (~6.5 years follow-up) and the DCCT-EDIC, with extended follow-up to 14 years, have demonstrated a reduction in CAN prevalence in individuals with type 1 diabetes with intensive glycaemic management (intensively treated arm vs conventional treatment arm: 28.9% vs 35.2%) [57–59]. However, the evidence regarding the prevention of CAN in type 2 diabetes is weaker. Notably, the Steno-2 trial revealed that optimising glycaemic management within a multifactorial risk factor reduction approach can contribute to CAN prevention, albeit this study included a relatively small cohort (*n*=160) [60].

### Weight loss and diet and exercise interventions

**Fig. 3:**
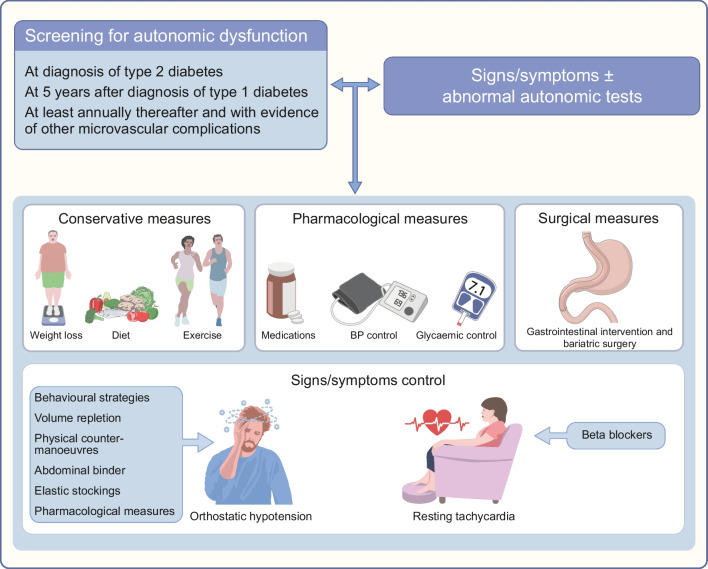
Overview of the management of CAN. CAN is diagnosed through screening or symptom evaluation. Its management focuses on symptomatic control (e.g. orthostatic hypotension and resting tachycardia) and strategies to delay progression or minimise cardiovascular risk factors (i.e. conservative, pharmacological and surgical measures) [7, 42, 43, 70–72, 110]. This figure is available as part of a downloadable slideset

Weight loss, along with dietary and exercise interventions, has been shown to enhance cardiac autonomic function [61]. A systematic review of exercise-based interventions demonstrated improvements in cardiac autonomic function (15/18 studies) in type 2 diabetes [62]. A combined diet and exercise intervention as part of a lifestyle programme in the Diabetes Prevention Program (DPP) demonstrated improvement in HRV, which was accompanied by a 58% reduction in the incidence of diabetes [63]. More recently, McGee et al have demonstrated significant improvements in HRV indices following a 10 week medical weight loss programme combined with aerobic exercise [64]. Although improvements in HRV and baroreflex sensitivity have been observed with lifestyle changes, conclusive evidence of regression, measured through CARTs, remains elusive. Hence, while lifestyle interventions may positively influence autonomic indices, definitive regression of CAN remains unattainable. An overview of the management of CAN is provided in Fig. 3.

### Bariatric surgery and autonomic dysfunction

The evidence regarding the effects of bariatric surgery on diabetic autonomic dysfunction is limited. While the STAMPEDE trial [65] highlighted that bariatric surgery can significantly improve components of the metabolic syndrome and hyperglycaemia, its impact on diabetic neuropathy is more complex. This complexity is primarily due to the common occurrence of deficiencies in micronutrients, which may counteract the beneficial effects of weight loss [66]. However, Casellini et al reported improved HRV indices in 70 participants 24 weeks following bariatric surgery [67] and Al Nou'mani et al found improvement in overall cardiac autonomic function after bariatric surgery in a meta-analysis [68]. Adequately powered, long-term observational and mechanistic studies are required to investigate this further.

### Metformin

Improvements in HRV indices have been demonstrated with metformin [69]. However, most people with type 2 diabetes are initiated with metformin from the outset.

### ACE inhibitors and angiotensin II receptor blockers

ACE inhibitors and angiotensin II receptor blockers are the first-line drug class of choice for hypertension and microalbuminuria in diabetes and may have a beneficial role in preventing CAN. Quinapril has been shown to enhance parasympathetic activity in individuals with diabetic autonomic neuropathy [70], and a recent extensive retrospective study (*n*=7464) suggested that pharmacological inhibition of the angiotensin system provides benefit in preventing peripheral neuropathy linked to type 2 diabetes, with potential implications for CAN, although further research is needed [71].

### β-blockers

β-blockers are an important therapy in CAN because of the high resting heart rate, which impairs diastolic coronary artery filling. β-blockers serve to lower the resting heart rate and improve HRV measures by modulating the sympatho-vagal balance [23]. They also improve diastolic coronary artery perfusion. Several large cohort studies including TRANSCEND, ONTARGET and BEAUTIFUL have shown adverse cardiovascular outcomes with a higher resting heart rate; thus, a reduction in heart rate may reduce cardiovascular risk in CAN [72, 73]. Bisoprolol has been shown to improve several HRV measures of parasympathetic activity in people with heart failure [74].

### Statins

Statins are a cornerstone in the management of dyslipidaemia in both type 1 and type 2 diabetes. Experimental data have shown the positive effects of fluvastatin on sympathetic cardiac innervation [75]. Studies have also shown that increased statin exposure reduces the odds of developing polyneuropathy [76], while Davis et al reported a dose-dependent reduction in polyneuropathy incidence in an observational study [77]. However, other studies have shown a neutral effect [78].

### Alpha-lipoic acid

Alpha-lipoic acid is a scavenger of free radicals and reduces oxidative stress driven by hyperglycaemia [79]. The ALADIN I and ALADIN II studies showed beneficial effects of alpha-lipoic acid on peripheral neuropathy and nerve conduction [80, 81]. Oral treatment with 800 mg/day alpha-lipoic acid for 4 months improved cardiac autonomic dysfunction, demonstrated by improvements in HRV, in type 2 diabetes [82]. However, another RCT did not demonstrate significant improvement in HRV with oral alpha-lipoic acid after 24 weeks of treatment [83]. Alpha-lipoic acid is incorporated into German guidelines for symptomatic diabetic neuropathy and the ADA suggests that it might have a role in the treatment of painful diabetic peripheral neuropathy [84, 85]. However, given the limited and inconclusive results on the effects of alpha-lipoic acid on autonomic cardiac indices, there are no recommendations in international guidelines regarding the use of alpha-lipoic acid for CAN.

## Newer diabetes therapies that may modulate the cardiac autonomic system

There are no established disease-modifying treatments for CAN. As such, newer therapies used in the treatment of diabetes have been investigated for their effects on the autonomic nervous system.

### Sodium–glucose cotransporter 2 inhibitors in type 2 diabetes

Numerous cardiovascular outcomes trials have demonstrated the positive impact of sodium–glucose cotransporter 2 inhibitors (SGLT2is) on cardiovascular outcomes. A meta-analysis (25 placebo-controlled and nine active-controlled RCTs) demonstrated a reduced risk of sudden cardiac death with SGLT2is compared with control (OR 0.72, 95% CI 0.54, 0.97; *p*=0.03) [86]. SGLT2is appear to mediate cardiovascular benefits through mechanisms extending beyond glycaemic management, with putative direct/indirect effects on the autonomic nervous system [87]. Recently, our real-world data study has mirrored outcomes from RCTs with SGLT2is, glucagon-like peptide-1 receptor agonists (GLP-1RAs) or combination therapy, with all three conferring mortality and cardiovascular protection in individuals with type 2 diabetes over 5 years [88].

Although the effect of SGLT2is on cardiovascular outcomes is well-established, there is conflicting evidence on their direct impact on autonomic dysfunction. Dapagliflozin has been shown to reduce noradrenaline (norepinephrine) expression in the kidney and improve renal haemodynamics independently of its effect on glycosuria [87, 89]. SGLT2is have also demonstrated consistent BP reduction in clinical trials. Compared with placebo, empagliflozin demonstrated a modest reduction in BP in the EMPA-REG trial (*n*=7020) [87]. Notably, there was no increase in compensatory heart rate, suggesting a possible inhibitory effect on sympathetic activity. However, direct tests of autonomic dysfunction were not performed in this study [90]. The EMBODY RCT of empagliflozin vs placebo evaluated cardiac sympathetic and parasympathetic nerve activity in individuals with type 2 diabetes (*n*=105), 2 weeks after acute myocardial infarction, finding improvement in most primary outcomes in the empagliflozin group but without intergroup differences [91]. However, the study was limited by its small sample size and limited exposure to SGLT2is. The SCAN study demonstrated that individuals with type 2 diabetes taking SLGT2is had improved cardiac autonomic function and reduced vasovagal syncope recurrence (which was associated with indexes of cardiac denervation) [92]. Other studies have reported a lower risk for arrhythmia, indicative of an effect on the autonomic nervous system [86]. However, the EMPA-HEART study (*n*=66 individuals with type 2 diabetes with established coronary artery disease) found no significant difference in HRV parameters between empagliflozin and placebo [93]. Additionally, a meta-analysis of pooled data from four RCTs (*n*=247 participants) demonstrated no effect of SGLT2is on autonomic imbalance [94]. The results of the last two studies should be interpreted in the context of the small sample sizes, with these studies likely being underpowered. Although the direct impact of SGLT2is on autonomic neuropathy remains to be demonstrated, given the elevated cardiovascular risk in people with CAN, SGLT2is are a crucial component of a multifaceted approach to reducing this risk.

### Glucagon-like peptide 1 receptor agonists in type 2 diabetes

GLP-1RAs are known to increase the heart rate and potentially reduce HRV [1]. Moreover, GLP-1RAs have demonstrated positive outcomes with regard to cardiovascular mortality risk [95]. In a recent meta-analysis, Greco et al confirmed an increase in heart rate but found no alteration in sympatho-vagal balance with chronic use of GLP-1RAs in individuals with diabetes [96]. Jaiswal et al reported no superiority in terms of CARTs or HRV following treatment with exenatide compared with insulin over 18 months in a small randomised trial (*n*=46) [97]. Recently, an observational study over 3 months found that GLP-1RAs (semaglutide/dulaglutide) improved nerve size and nerve morphology, reduced the severity of neuropathy and improved sural sensory nerve conduction amplitude, suggesting a direct structural improvement to the nervous system [98]. While evidence of the direct impact of GLP-1RAs on CAN is limited, similar to SGLT2is, their potential to ameliorate cardiovascular mortality risk should be considered. Their use should be avoided in the presence of gastroparesis because of their association with slowed gastric emptying and worsening gastroparesis [32].

### GLP-1RAs and SGLT2is in type 1 diabetes

Laursen et al found that 4 weeks’ treatment with empagliflozin did not significantly affect HRV or CARTs in individuals with type 1 diabetes [99]. Notably, the study's limitations included the small number of participants, the absence of an active control group and the short exposure period of 4 weeks to empagliflozin, with the authors concluding that further RCTs are needed. We recently published a real-world data study of SGLT2is and GLP-1RA in type 1 diabetes [100]. We demonstrated that both SGLT2is and GLP-1RAs have potential benefits as adjunctive agents in type 1 diabetes in terms of reno-vascular outcomes. RCTs are needed to establish if there are any real benefits.

## Future research

Further work is essential to identify the molecular and cellular mechanisms involved in the pathogenesis of CAN in terms of both development and progression, with a greater understanding needed of mitochondrial dysfunction and genetics/genomics. Tissue, skin and blood samples analysed through a multifaceted ‘omics’ approach—including genomics and proteomics—may identify predictors of CAN development. Additionally, spatial transcriptomics of autonomic nerve fibres in sweat glands could provide further insights [101]. Exploring these mechanisms in cohorts developing early CAN in prediabetes, which progresses to overt CAN in type 2 diabetes, may provide a fundamental understanding of the pathophysiology of CAN. Together, these methods could help identify individuals at risk of CAN and guide future personalised treatments.

To further understand the pathophysiology of CAN, the role of obstructive sleep apnoea, which is very common in people with type 1 and type 2 diabetes, also needs to be evaluated, especially as obstructive sleep apnoea is reversible and is associated with autonomic neuropathy in people without diabetes. Additionally, the combination of lifestyle and pharmacological interventions, in particular early in the natural history of CAN, should be evaluated in cohort studies or clinical trials. This should include the use of newer therapies such as SGLT2is and GLP-1RAs combined with dietary modification to investigate the prevention of progression (or even reversal) of CAN.

Digital health technologies and wearable devices have been shown to be accurate in the diagnosis of cardiovascular conditions such as atrial fibrillation. If CAN screening is introduced and provides valuable real-time data on autonomic function, remote monitoring using wearable technology such as the WHOOP band, which has been shown to provide reliable HRV measurements [102], may negate/reduce the burden on screening services. Future strategies to implement CAN screening as part of routine practice may include using retinal imaging, specifically CCM, or 12 lead ECGs in conjunction with artificial intelligence-based models, the latter of which we have recently demonstrated has good-to-excellent diagnostic ability with regard to CAN [103].

## Conclusion

The prevalence of CAN is expected to rise in line with the projected increase in the number of people with diabetes, and the associated mortality risk underscores the importance of prevention and early diagnosis. Although there are currently no established pharmacological interventions targeting its pathophysiology, evidence suggests that stringent glycaemic management and lifestyle modifications, along with the mitigation of risk factors, can partially ameliorate indices of CAN. Exploring the pathophysiology of CAN and evaluating novel therapies are crucial for advancing our understanding and developing potential treatment options for this condition.

## Supplementary Information

Below is the link to the electronic supplementary material.

Slideset of figures (PPTX 508 KB)

## Abbreviations

ABPM: Ambulatory BP monitoring
CAN: Cardiovascular autonomic neuropathy
CART: Cardiac autonomic reflex text
CCM: Corneal confocal microscopy
GLP-1RA: Glucagon-like peptide-1 receptor agonist
HRV: Heart rate variability
QTc: Corrected QT interval
SGLT2i: Sodium–glucose cotransporter 2 inhibitor
