# Supplementary material for: Cardiovascular autonomic neuropathy in diabetes: an update with a focus on management
Source: Diabetologia. 2024 Aug 9;67(12):2611–25. doi: 10.1007/s00125-024-06242-0 (PMC11604676; doi:10.1007/s00125-024-06242-0)
Supplement: Supplementary file 1 — Slideset of figures (PPTX 508 KB) [file 125_2024_6242_MOESM1_ESM.pptx]

## Slide 1
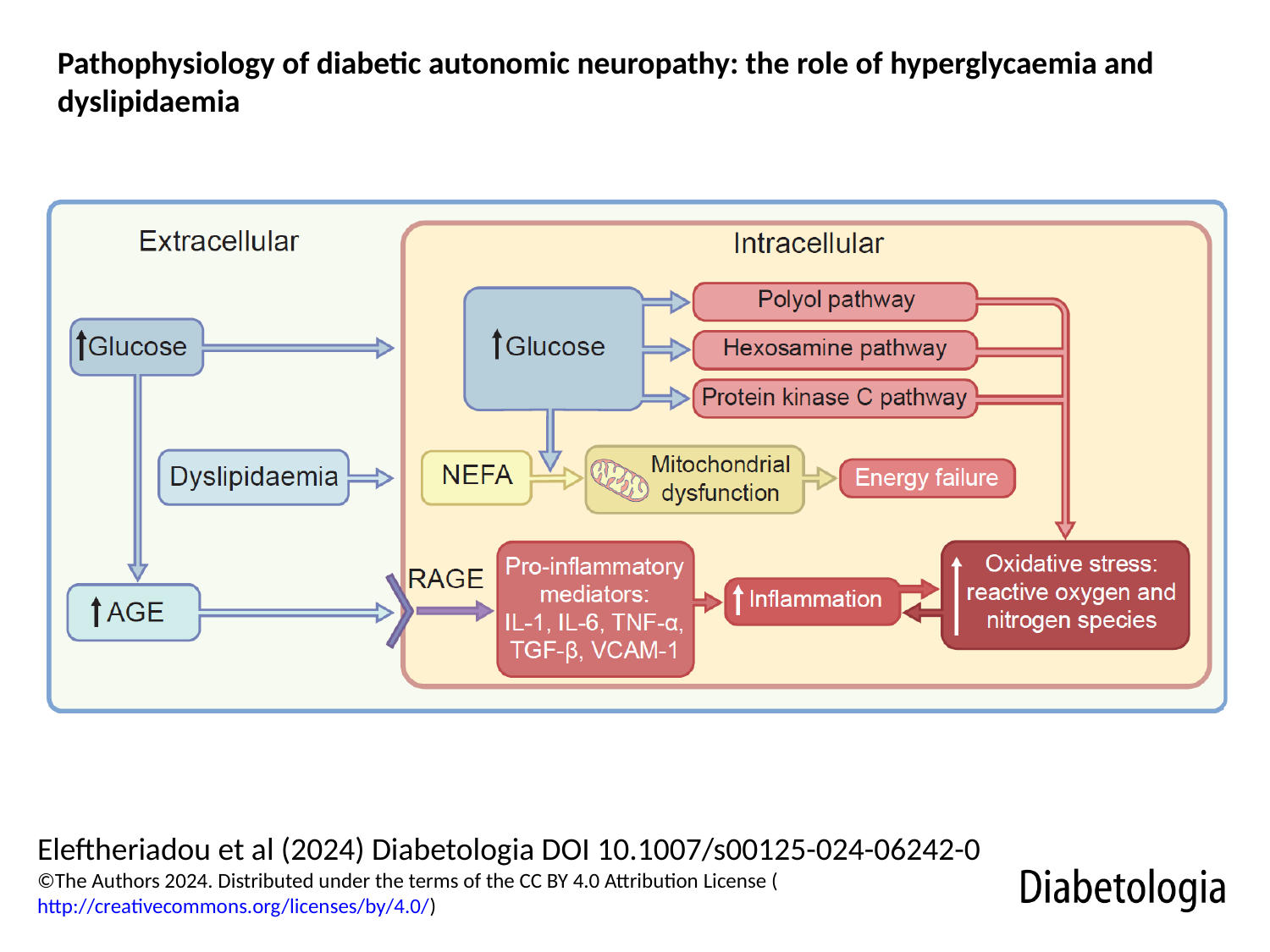

Pathophysiology of diabetic autonomic neuropathy: the role of hyperglycaemia and dyslipidaemia
Eleftheriadou et al (2024) Diabetologia DOI 10.1007/s00125-024-06242-0
©The Authors 2024. Distributed under the terms of the CC BY 4.0 Attribution License (http://creativecommons.org/licenses/by/4.0/)

## Slide 2
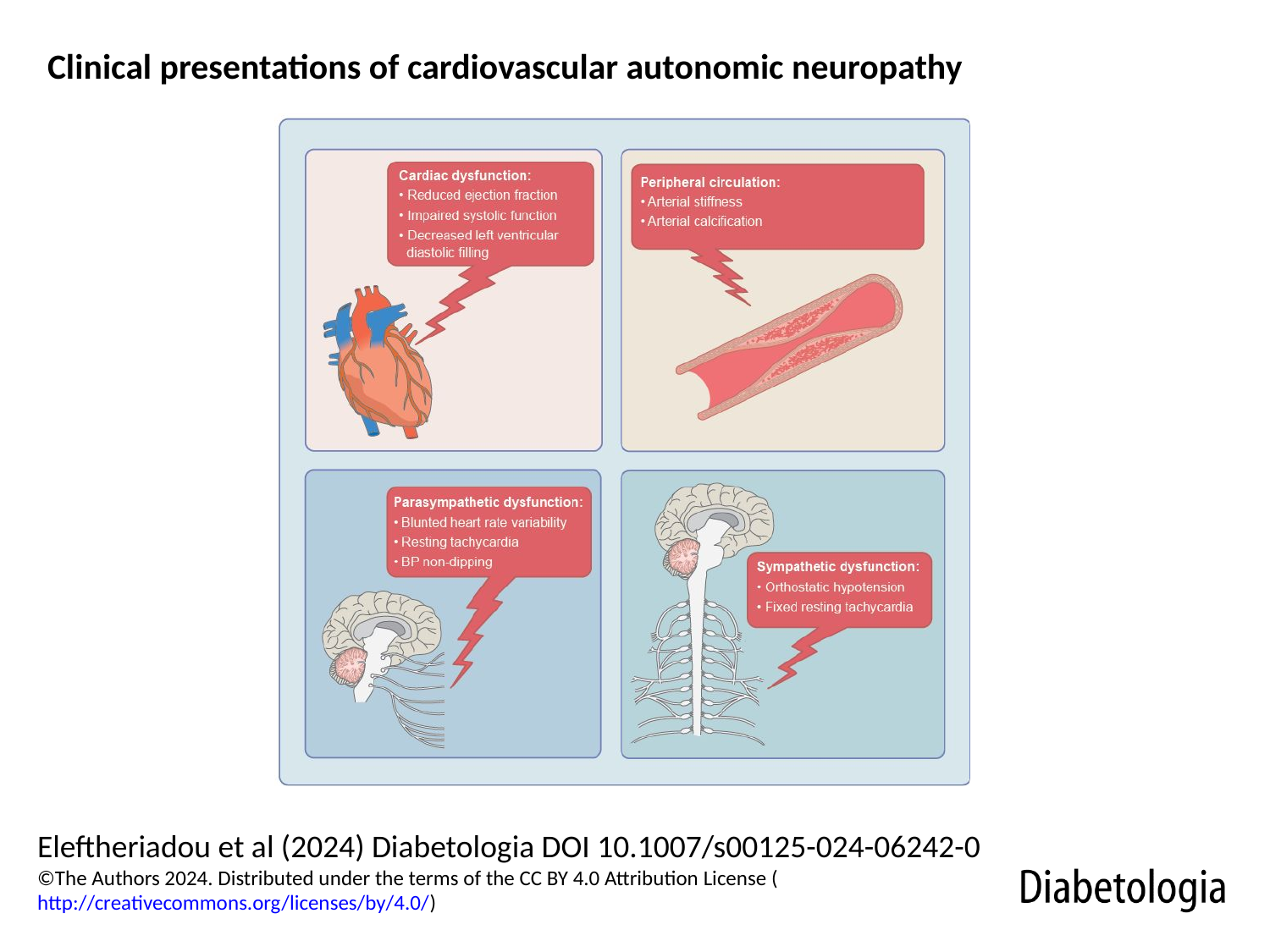

Clinical presentations of cardiovascular autonomic neuropathy
Eleftheriadou et al (2024) Diabetologia DOI 10.1007/s00125-024-06242-0
©The Authors 2024. Distributed under the terms of the CC BY 4.0 Attribution License (http://creativecommons.org/licenses/by/4.0/)

## Slide 3
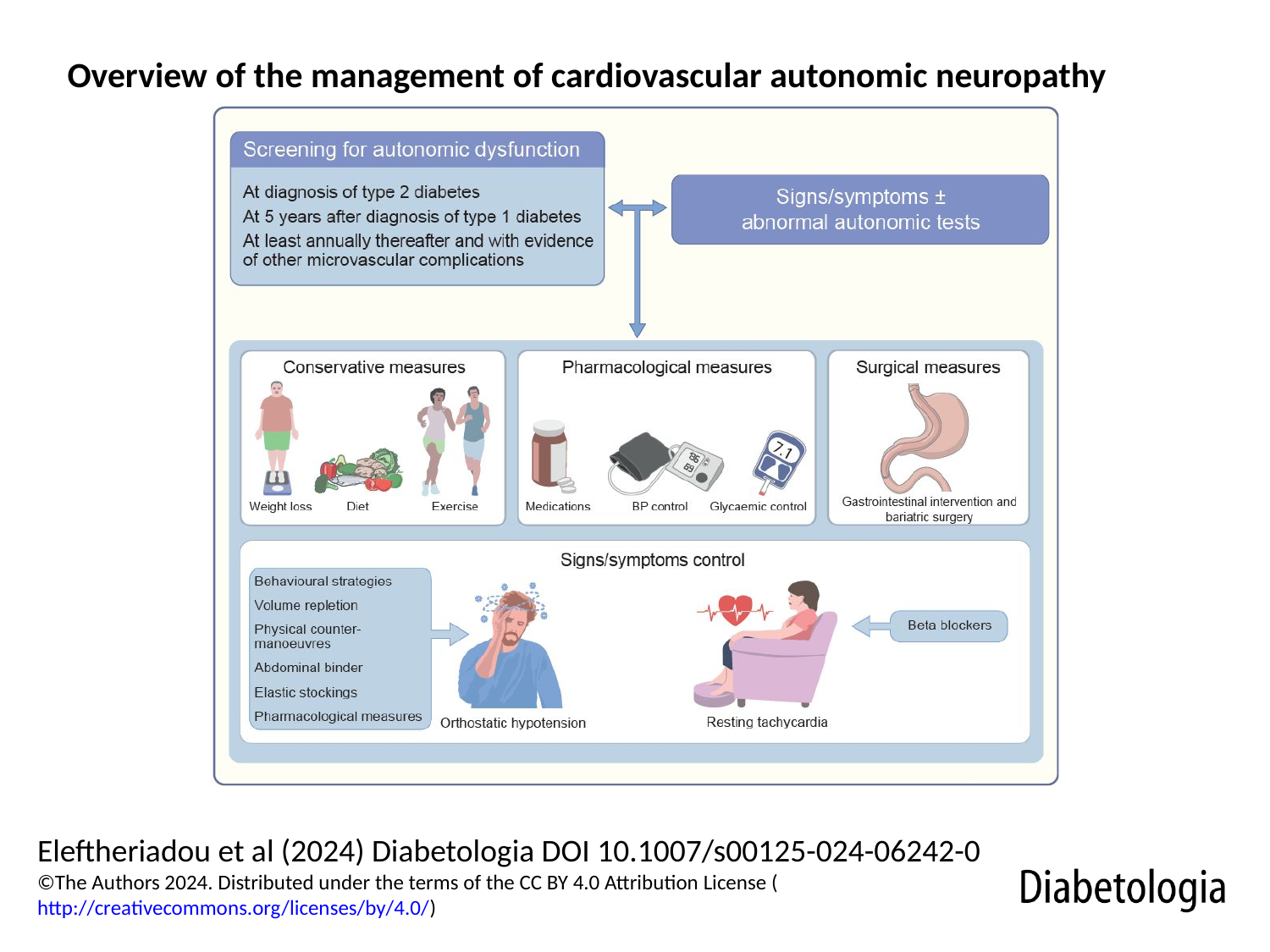

Overview of the management of cardiovascular autonomic neuropathy
Eleftheriadou et al (2024) Diabetologia DOI 10.1007/s00125-024-06242-0
©The Authors 2024. Distributed under the terms of the CC BY 4.0 Attribution License (http://creativecommons.org/licenses/by/4.0/)
